# 

**Published:** 1907-12-07

**Authors:** 


CONTENTS.

PAGE

**The Medical Inspection of School Children** - - 251

**A Bill for the Prevention of Premature Burial** - 252

**Annotations—**
Royal College of Surgeons Annual Meeting—Pharmacopœial Revision—Reform in Medical Nomenclature - - - - 253

**Medical Opinion and Movement** - - - - - - - 254

**Hospital Clinics—**
Senile Respiratory Disorders. By Sir R. Douglas Powell, Bt., K.C.V.O., M.D. - - - - - - - - - 255
Gravel and Stone. Clinical Remarks on their Formation and Treatment. By Reginald Harrison, F.R.C.S. - - - - 257

**Illustrative Cases—**
The Persistence of Typhoid Bacilli in Organs for Years - - 259

**Clinical Methods—**
Stains for Fat - - - - - - - - - - - - - 260

**Points in Surgery—**
The Treatment of Fractures from a Common-sense Point of View - - - - - - - - - - - - - 261
Osteo-Arthritis - - - - - - - - - - - - - 262

PAGE

**Laryngology and Rhinology—**
Local Applications to the Upper Air Passages - - - - - 263

**Dermatology—**
The Use and Abuse of Soap - - - - - - - - 264

**The General Practitioner's Column—**
Treatment of Pulmonary Phthisis - - - - - - - 265

**Practical Notes on Diagnosis and Treatment** - 266

**Therapeutics—**
Prescriptions and Dispensing - - - - - - - 267

**The Book World of Medicine and Science** - - 268

**Hospital Administration—**
The Royal Free Hospital - - - - - - - - - 269
The Hampstead General Hospital - - - - - - - 270
The Poplar Hospital for Accidents - - - - - - - 272
The Medical Inspection of Children in Public Elementary Schools - - - - - - - - - - - - 273
News and Coming Events - - - - - - - - - - 274

**Nursing Administration—**
The Registration of Nursing Homes - - - - - - 275

**Editor's Letter-Box—**
Pensions and the Medical Profession - - - - - - 276
The Graduates' Medical Schools - - - - - - - 276

